# Priorities of China’s participation in global malaria elimination: the perspective of malaria endemic countries

**DOI:** 10.1186/s40249-022-00970-4

**Published:** 2022-04-20

**Authors:** Yan Xie, Jie Wang, Yinuo Sun, Xuedan Ke, Zheng Xie, Jun Cao, Yangmu Huang

**Affiliations:** 1 Peking University School of Public Health, Beijing, 100191 China; 2 Institute for Global Health and Development of Peking University, Beijing, 100191 China; 3 Key Laboratory of National Health and Family Planning Commission on Parasitic Disease Control and Prevention; Jiangsu Provincial Key Laboratory on Parasite and Vector Control Technology; WHO Collaborating Centre for Research and Training on Malaria Elimination, Jiangsu Institute of Parasitic Diseases, Wuxi, 214064 China; 4 Ministry of Education Key Laboratory of Carbohydrate Chemistry and Biotechnology, School of Biotechnology, Jiangnan University, 1800 Lihu Avenue, Wuxi, 214122 China; 5 Center for Global Health, School of Public Health, Nanjing Medical University, Nanjing, 211166 China

**Keywords:** Malaria elimination, Aid effectiveness, China

## Abstract

**Background:**

Malaria is one of the major diseases affecting global health, while progress in malaria control and elimination has stagnated in some endemic countries. China has been certificated malaria free by World Health Organization in 2021, and will get more involved on global malaria elimination. Further discussion is needed on how to collaborate with the malaria endemic countries and provide effective help. This study was to investigate the perceptions of malaria endemic countries on China’s contribution to global malaria elimination and to lay a foundation for further action.

**Methods:**

Semi-structured interviews were conducted with key informants including national malaria project managers and technicians from malaria endemic countries. Thematic framework approach was used to analyze the data.

**Results:**

Malaria endemic countries now face challenges in insufficient funds, technique, products, public health systems and inadequacy of international assistance. They hold a positive attitude towards cooperation with China and identified experience and technique exchange, personnel training, system building and scientific research cooperation as prioritized areas.

**Conclusions:**

China could make full use of its own advantages in technique transfer, health system improvement, information system construction, and health human resource training and take an active part in global malaria elimination.

## Background

As one of the major infectious diseases, malaria affected 241 million people and caused 627,000 deaths in 85 malaria endemic countries in 2020 [1]. Though some countries have entered the phase of malaria elimination, progress in malaria control has recently been stagnated due to limited resources [2]. Some countries and regions are still confronted with a high burden of malaria, mainly in Africa and South Asia. It is estimated that the World Health Organization (WHO) African Region accounted for about 82% cases and 95% deaths globally, followed by the WHO South Asia Region (10% of cases and 2% of deaths). Besides, despite relatively low incidence and death rate, malaria control in the WHO Western Pacific region is characterized by stagnation [2]. Especially in 2020, the incidence and death rate in these three regions have all increased, which was associated with disrupted diagnosis and treatment services during the COVID-19 pandemic [3, 4]. WHO has indicated that endemic countries tend to hold weak health system. It’s difficult for them to adapt the global strategy to local context, as well as balance new health emergencies and malaria during pandemic. What’s more, resistance to effective antimalarial drugs, insufficient fund and so on have posed an added challenge to malaria control in these regions [5–7]. Thus, coordinated global action is urgently needed to incorporate new inputs and contributions from partners in funding, products, diagnosis, treatment, technique, and surveillance [8].

China, once a country suffering from a severe malaria epidemic, has worked for decades to realize the goal of malaria elimination by 2021 [9]. With rich experiences in malaria elimination, China has formulated strategies and developed many high-quality and cost-effective anti-malarial products [10]. It is believed that China could play a significant role in global malaria elimination, for its contribution of malaria elimination as a country with one-fifth of the world’s population, its experiences such as the formulation of “1-3-7” norm and its great discovery of artemisinin and so on [5]. In the context of increasing demand for international cooperation, China is willing to share the experience, technology and products with other epidemic countries along their way towards malaria elimination.

Malaria control has been a traditional key field of China’s health development assistance [11]. With the recognition of China’s malaria elimination, international collaboration on malarial control has been more prioritized recently. For example, the China–Africa Cooperation Forum has made malaria control cooperation a priority [12]. During the past few decades, China aided malaria-endemic countries in various forms, mainly including donation of anti-malarial drugs, establishing antimalarial centers, carrying out training and intervention programs [11], the effectiveness of which has been widely concerned. Both *Paris Declaration* and *Busan Declaration* have attached great importance to the role of recipient countries’ participation and ownership in improving the effectiveness of aid, so implementing approaches need to be tailored to country-specific situations and needs [13, 14]. China’s participation in global malaria elimination should base on full understanding of and respond to the needs of beneficiary countries. By interviewing managers and experts from other malaria endemic countries, this study investigates the views of recipient countries on how China could participate in the global malaria elimination, so as to provide a basis for China to develop strategies for further action.

## Methods

### Respondents

Individuals who had been responsible for or involved in malaria control in malaria endemic countries were considered as key informants for this study. They were divided into three groups: (1) officers of health departments, (2) managers of malaria programs and (3) technicians in malaria-endemic countries.

### Sampling strategy

A survey was conducted on the participants of Seminar on Malaria Control for Developing Countries held by Jiangsu Institute of Parasitic Diseases. Purposive sampling was used to select malaria-endemic countries in different WHO regions. Based on the demographic characteristics of seminar participants, a total of three regions including 13 countries were involved in this study, and 1–3 respondents for each country and a total of 18 respondents were selected (Table 1).

**Table 1:** Country distribution and number of respondents

WHO Region	Malaria-endemic countries (number of respondents)
African	Ethiopia (1), Gambia (1), Ghana (1), Malawi (2), Sudan (1), Zambia (3)
South-East Asia	Nepal (1), Bangladesh (1), Myanmar (1)
Western Pacific	Malaysia (1), Cambodia (2), Laos (2), Vietnam (1)

### Data collection

Semi-structured interviews were used. Respondents were interviewed according to the topic guide in a quiet and independent room. With the consent of the respondents, interviews were tape recorded. All interviews were conducted in English and were based on the principle of information saturation. The first respondent was selected according to survey subject and the information was summarized by constant comparative method. The second respondent was selected by maximum difference scaling. Above steps were repeated until no more new information was available, and the investigation was complete.

### Data analysis

Interviews were transcribed verbatim into Word files. The transcribed data were read and checked carefully for several times to ensure the accuracy. Then the thematic framework analysis was applied to analyze the data. Themes and subthemes were developed as follows (Table 2) after multiple rounds of discussion by the research group. According to the coding table, the interview material was coded and key information was extracted to draw a conclusion.

**Table 2:** Thematic framework

Theme	Subtheme
1 Major challenges in malaria elimination faced by the countries	1.1 Shortage of funding 1.2 Insufficient technical personnel 1.3 Challenges on vector control 1.4 Lack of anti-malaria products 1.5 Challenges on universal health coverage 1.6 Unsound malaria surveillance system
2 Problems in existing forms of international assistance	2.1 Challenges in Global Fund’s support 2.2 Problems in WHO’s technical assistance
3 Priorities of China’s participation	3.1 Experience and technique sharing 3.2 Health human resource training 3.3 Public health system building 3.4 Scientific research cooperation

### Quality control

We conducted systematic training for the interviewers before data collection. As for data analysis, the data were transcribed by two authors to ensure the accuracy. Thematic framework was developed after the discussion and consultation with experts. The coding process was conducted by two persons with comparation and revision, and cross checking was conducted by another author to ensure no codes were missed.

## Results

### Participants

A total of 18 key informants from different malaria-endemic countries were interviewed, including 16 males and 2 females, aged between 30 and 50 years. Types, professional affiliation and number of respondents were summarized in Table 3.

**Table 3:** Type and number of respondents

Types of respondents	Affiliation	Number
Officers in Health Departments	National office	5
	Regional office	3
Managers of national malaria programs	Provincial and regional office	2
	District office	1
Technicians and professionals	Research institutions/universities	5
	Hospitals/health facilities	2

### Major challenges in malaria elimination faced by the countries

#### Shortage of funding

The Global Fund to Fight AIDS, Tuberculosis and Malaria (hereinafter referred to as Global Fund) was the most important source of funding commonly mentioned by respondents. Respondents in Laos revealed that the Global Fund has reduced its budget and that *“Global Fund played the dominant role 5 years ago, but now the government takes its place”* (antimalarial programme manager). Bangladesh respondent said funding was relatively insufficient based on its large population. Malaysia respondent denoted that concerns and investments in malaria were limited due to the needs for other infectious diseases such as dengue fever, as well as chronic diseases. Many recipient countries of the Global Fund mentioned that once malaria burden fell or progress stalled, attention and focus would disappear, further investment would be difficult to obtain, therefore the sustainability of funding failed to be assured.

#### Insufficient technical personnel

In general, both quantity and quality of health workers in malaria-endemic countries are insufficient. Qualitatively, certain lack is identified in the ability of field workers. And in terms of quantity, there is no adequate high-level talents and laboratory technicians. In African and South-East Asian countries, field work is usually undertaken by volunteers, whose ability and motivation to work are relatively low. Besides, entomologists and senior experts of public health in these countries are deficient. Respondents of Ghana said that universities in Ghana were unable to provide such subjects education, while professionals trained abroad often chose to work in other countries for better working environment and salary. Many respondents also mentioned the lack of domestic training mechanism, and the challenge to attract and retain technical personnel who received foreign training.

#### Challenges on vector control

Insecticide-treated mosquito net/long-lasting insecticidal net (ITN/LLIN) and indoor residual insecticide spraying (IRS) are two core vector control interventions. However, in Africa, it is very common that the coverage rate of mosquito nets is high but utilization rate is low, for which reasons can be summarized as follows: no bed for hanging mosquito nets; no accompanying education and instruction; wide spread of rumors, superstition and other adverse factor due to low literacy rate in rural areas; poor user experience due to bad smell of new mosquito nets.

And as for insecticide spraying, volunteers must take along pesticides and tools to spray everywhere. It is hindered by unsuitable housing conditions, insufficient insecticides, insufficient health workers and poor transportation, et al. Respondents also noted that failure of project management to adapt to local conditions also reduced spraying effectiveness. For instance, in tropical Africa, spraying must be finished before rainy season, but the right time is often missed because of funding and supplies delay.

#### Lack of antimalarial products

Some respondents from Africa also mentioned that there were no adequate supplies of affordable and high-quality antimalarial products. Respondent from Sudan said that the domestic supply chain of diagnostic preparations, drugs, vaccines and vector control tools was imperfect, with varying degrees of invalidity and waste. In the production and supply of antimalarial products, insufficient involvement of private sectors, serious bureaucracy and corruption in public sectors and nonstandard product procurement have resulted in high price and difficult access. In African countries, drug research and development as well as local manufacturing capacity is weak, the antimalarials exposed to the respondents are all imported from different countries. The quality of antimalarial drugs from different sources varies greatly, and the therapeutic effect cannot be guaranteed.

#### Challenges on universal health coverage (UHC)

In malaria-endemic countries, unequal access to health education, products, and interventions poses a huge challenge to the elimination of malaria. Many African countries indicated that the distance between health facilities in rural areas and between facilities and hospitals made it difficult to ensure vector control coverage or timely transport of severe malaria patients. A large number of residents in South-East Asian countries live and work in remote mountainous areas, which makes interventions inaccessible to high-risk population. Border malaria due to population movements as cross border, migrants, abroad and uncontrolled seasonal workers is also a common challenge in this region. Imported malaria has gradually become a difficulty in malaria control.

#### Unsound malaria surveillance system

Malaria surveillance system in malaria-endemic countries is poorly developed, of which the weaknesses arisen from community level. Respondents of Ethiopia stated that “*real-time surveillance must be carried out at the community level, but it has not been implemented properly*” (officer in health ministry). The current lack of initiative and credibility in community case reporting is not confined to areas of high transmission. According to respondents of Myanmar, *“most tests in low-transmission areas are negative, which negatively affect motivation of volunteers and diagnostic accuracy.”* (manager of infectious disease project). Respondents of Bangladesh and Vietnam said they didn’t have essential network, computers and mobile phones in communities needed for real-time reporting. In Vietnam, 2-3-7 strategy instead of 1-3-7 norm is used, since it is not possible to complete a report by telephone or text message within 1 day without modern equipments. Active case detection (ACD) is required in areas or populations with limited access to health care to eliminate malaria, whereas respondents of Malawi stated *“now we can only do passive case detection”* (public health professional, male, 38). These countries also have difficulty in managing and using the surveillance data, as one respondent mentioned that “*we just collect data, we’re not using it*” (district health officer).

### Challenges in existing forms of international assistance

#### Challenges in Global Fund’s support

Most respondents regard Global Fund as large-scale investment, which provides a great number of mosquito nets, insecticides, antimalarials, rapid diagnostic test agents and other products, but there are problems in the way of money distribution and management. Managers of malaria projects pointed out that there were gaps between planning and the actual needs due to inadequate research. They also pointed out that money from Global Fund usually specified the usage without meeting the real need. In addition, funding application process usually takes a long time, and cannot remain predictable and sustainable, resulting in delayed intervention or suspended programme. Zambia and other African countries complained about the internal management of the Global Fund, arguing that a large amount of money was consumed by unnecessary meetings and activities.

#### Problems in WHO’s technical assistance

Technical support in malaria-endemic countries is mainly provided by the WHO. According to respondents of African countries, the WHO’s documents were mechanically copied by most countries and there was a lack of coordination and guidance from WHO. They also indicated that frontline health workers were not involved in making national malaria strategic plan, so there was lack of practical feedback. One respondent said *“Some of the challenges proposed by national malaria strategic plan are real, but some are hypothetical without evidence.”* (district-level health officer). Moreover, project implementers in recipient countries lack ownership and are often forced to stop current work and quickly adopted the new instruction. Respondents of Sudan and Ghana also pointed out that WHO usually tended to focus on areas with high disease burden, but ignored areas with low disease burden, which was not conducive to achieving the goal of malaria elimination.

### Priorities of China’s participation

#### Experience and technique sharing

Experience and technique sharing are what respondents of all countries expect most from China. In their opinion, China’s own successful experience in malaria control makes its advantage compared to other partners or leaders in global malaria elimination. Respondents stated that compared to funding support, *“sharing your experience and techniques is more valuable to us”* (manager of infectious disease programme). Many respondents believed that China’s success could be replicated in their countries since they are all developing countries with similar history of malaria epidemic. Many countries entering the stage of malaria elimination like Vietnam showed their interest in China’s “1-3-7” norm, while Malawi expect China to help the implementation of mass drug administration (MDA). Respondents said that current technical strategies fail to receive specific guidelines from WHO, and China could provide rich experience in developing and applying appropriate strategies.

#### Health human resource training

Capacity building is urgently needed in malaria-endemic countries and there is a great need for health human resources training. As for who should be trained, respondents who works as laboratory staffs said they needed to train more technicians to operate microscopes and PCR. District-level health officers indicated a need to train project executors to think in the context of public health and conduct epidemiological investigation to provide evidence for decision-making. Also, one respondent believed that to improve the efficiency of training, high-level officers should be trained first, for they had influence in policy making to promote the malaria control framework in the country. As for the form of training, respondents suggested both inviting Chinese experts to their countries and sending personnel to China were appropriate. But they also mentioned that providing lectures in their country would be more beneficial and cost-effective than sending personnel to China, especially for mass personnel training. In addition, some respondents mentioned that there were language barriers in the current training held by China, and they expected better communication in future trainings.

#### Public health system building

The needs of public health systems building in malaria-endemic countries mainly focused on disease control network, surveillance, emergency care and health information system. Respondent of Bangladesh said, *“we want support from China on how we can develop this excellent CDC network from national level to township level”* (surgeon). Respondents in Ethiopia said they needed China’s help with “*community-based real-time surveillance and response systems*”. Respondent from Sudan recommended the Chinese government to support them in emergency care so as to respond quickly to new cases (hospital director). Several respondents from Africa suggested that China help improve the utilization of health information because in their countries they have collected large amounts of data but had no the ability to analyze and use them properly.

#### Scientific research cooperation

There is a great need for malaria-endemic countries to collaborate with China in joint case diagnosis and research for difficult and complicated cases. Respondents mentioned that there are limited scientific research in their countries which makes it difficult to deal with complex and changing situation of the disease. One respondent stated that in his country, there was no specialized research institute for parasitic diseases, and China’s help for establishing such institute would increase their capacity to control and eliminate malaria. In the Greater Mekong Sub-region facing the challenge of artemisinin resistance, respondents of Cambodia, Vietnam and Laos mentioned they were willing to conduct relevant research in their countries which need additional support (deputy director of laboratory). Some respondents from African countries pointed out the limitations of the current vector control-based strategies, and expressed their hope to collaboratively carry out pilot projects to develop more localized strategies.

## Discussion

In the hope of increasing the participation and ownership of malaria-endemic countries in improving the effectiveness of aid, this qualitative study provides a comprehensive analysis of the current challenges to malaria control and elimination in malaria-endemic countries—and summarized the prioritized areas in which China could contribute to the global efforts.

In general, the results indicated that major challenges in malaria elimination faced by the countries fall in shortage of funding, technology and antimalarial products; unequal access to health education, products, and interventions; and unsound surveillance system. Nevertheless, malaria-endemic regions with various status quo are confronted with different urgent need. Specifically, in African countries, rely heavily on the multichannel imported anti-malarial product without guaranteed quality and effect due to inadequate research and development capacity. In addition, a greater concern is independently emerging in artemisinin resistance [15, 16]. Thus, for African countries with high malaria transmission, an immediate priority is to ensure the availability of effective anti-malaria products. While in South-East Asian countries entering elimination phase, the main challenge is the gap between their current capacity and what is needed for elimination, which requires upgraded surveillance and more specific diagnosis, etc. Goals and strategies in different stages of malaria control vary greatly [17]. China’s experiences accumulated during malaria control and elimination phases, could help classify the endemic areas by local conditions and provide specific assistance strategies accordingly.

Though concerted efforts have been made by countries and global partners, some challenges are manifested from current assistance, such as the lacking ownership of recipient countries for assistance programs. For a long time, the international aid system is dominated by donor countries, which is proven less effective [18]. Firstly, assistant priorities put up by donor countries sometimes diverge from the actual need. For example, the Global Fund promote the vector control, whereas reviews about mass distribution campaigns in Malawi show that a single intervention-based approach for vector control cannot have optimal impact in areas with high malaria transmission [19]. Effective and sustainable vector control can only be achieved through coordinated and integrated management, with improved resistance management and health education. Secondly, vertical programs that directly executed by international agencies or organizations are often difficult to integrate with weak local health systems. The International Association of National Public Health Institutions has demonstrated that in low-resource countries, country-led public health institutions are the best way to ensure the self-sufficient and sustainable systems over the long term [20]. Therefore, it’s crucial for donors to return the ownership of assistance programs to recipient countries, especially endow their participation in the development of evidence-based strategies.

The COVID-19 pandemic has presented additional challenges for malaria control. Although many donors have helped malaria-countries to maintain essential health services by providing greater flexibility in the allocation of disease specific funds, most endemic countries failed to maintain essential health services in the context of COVID-19. Service disruption extended to several aspects including the provision of interventions and surveillance. Firstly, the supply chain for treatments has experienced disruptions due to transport interruption domestic and overseas, leading to the poor accessibility of health commodities. Secondly, unsound surveillance was unable to cope with double pressures of COVID-19 and malaria, so the limitations on data have seriously challenging the patients tracing, diagnosis and treatment. COVID-19 pandemic further highlights the importance of malaria aid for these malaria endemic countries.

China’s experience in malaria elimination have been proved to be conductive in assisting countries with high malaria burden [21]. Given the urgency of providing targeted assistance for malaria endemic countries during COVID-19 pandemic and the priorities of China’s assistance identified in our study, we believe China could contribute mainly on technology, health system, information system, and human resources, et al. Specific recommendations are as follows: to promote the innovation and access of anti-malaria products, specifically to promote technology transfer and localized production of artemisinin-based drugs. To combine malaria interventions with the construction of health systems, help to build local three-level health service network so as to achieve sustainable development of assistance programmes. To provide technical support for building health information systems, and improving ability for surveillance and data utilization at all levels for better evidence-based decision-making. To carry out personnel training including laboratory operation training, project management training, as well as malaria relevant scientific cooperation through universities and research institutes. In addition to bilateral cooperation, China could contribute to multilateral South-South cooperation as well as regional and international cooperation. To strengthen cooperation with WHO and other aid agencies in joint efforts to mitigate disruptions to malaria services during the pandemic, conduct research based on the national conditions, and formulate targeted malaria intervention plans based on the national strategic plan.

This study interviewed with officers in health departments, managers of national malaria programs and technicians and professionals in malaria-endemic regions may increase the authority and reliability of the results. However, it was conducted prior to COVID-19 pandemic in 2018, so the challenges and problems facing endemic countries may aggravate in the context of pandemic. We may underestimate the seriousness of current situation and ignore some emerging threat in these regions. What’s more, due to the limited participants of the seminar, the selected areas did not cover all the endemic regions around the world such as Eastern Mediterranean region and region of Americas, and to some extent, the results were not representative of all the endemic regions.

## Conclusions

The increasing threats and challenges in funding, technology and human source have posed a tremendous obstacle for malaria endemic countries. China could take an active part in global malaria elimination, and cooperate with malaria endemic countries on including, but not limited to technique transfer, health system improvement, information system construction, and health human resource training. Possessing rich experience of achieving zero malaria cases from 30 million, China could promote the process towards malaria elimination in endemic countries, and also provide concerted assistance along with other international parties.

## Data Availability

All data generated or analyzed during this study are included in this article.
